# Molecular Detection of *Rickettsia* and Other Bacteria in Ticks and Birds in an Urban Fragment of Tropical Dry Forest in Magdalena, Colombia

**DOI:** 10.3390/life13010145

**Published:** 2023-01-04

**Authors:** Miguel Mateo Rodriguez, Angel Oviedo, Daniel Bautista, Diana Patricia Tamaris-Turizo, Fernando S. Flores, Lyda R. Castro

**Affiliations:** 1 Center of Genetics and Molecular Biology, Universidad del Magdalena, Santa Marta 470001, Colombia; 2 Research Group in Biodiversity and Applied Ecology (GIBEA), Universidad del Magdalena, Santa Marta 470001, Colombia; 3 Universidad Nacional de Córdoba (UNC), Córdoba 5016, Argentina; 4 Instituto de Investigaciones Biológicas y Tecnológicas (IIByT), Consejo Nacional de Investigaciones Científicas y Técnicas (CONICET), Córdoba 5016, Argentina

**Keywords:** ticks, birds, blood, ectoparasites, 16S meta-taxonomy

## Abstract

Birds are important hosts in the life cycle of some species of ticks. In Colombia, there are few eco-epidemiological studies of tick-borne diseases; the existing ones have been focused on areas where unusual outbreaks have occurred. This study describes the identification of ticks collected from birds and vegetation, and the detection of bacteria in those ticks and in blood samples from birds in an urban fragment of tropical dry forest in the department of Magdalena, Colombia. Bird sampling was carried out monthly in 2021, and 367 birds, distributed among 41 species, were captured. All collected ticks were identified as *Amblyomma* sp. or *Amblyomma dissimile*. The presence of rickettsiae in ticks collected from birds was evaluated by molecular analysis of the gltA, ompA and sca1 genes. 16S rRNA meta-taxonomy was used to evaluate rickettsiae in ticks collected from vegetation and in blood samples from birds. The presence of the species “*Candidatus* Rickettsia colombianensi” was detected in ticks from birds. Bacteria of the family Rickettsiacea was the most abundant in ticks collected from vegetation. Bacteria of the families Staphylococcaceae, Comamonadaceae and Pseudomonadaceae were prevalent in the samples of blood from birds. *Rickettsia* spp. was also detected in low abundance in some of the bird blood samples.

## 1. Introduction

Zoonosis refers to diseases that are transmitted from animals to humans, and they represent a serious threat to the health and well-being of people [1]. New zoonotic diseases are constantly emerging as human activity expands into new territories that contain natural foci of infection. Some of the main infectious agents involved include bacteria, viruses and fungi, among others [2].

Ticks are obligate hematophagous ectoparasites and some species can act as vectors of pathogenic microorganisms such as protozoa, bacteria and viruses [3,4,5]. They are cosmopolitan, mainly from warm climates [4], and are recognized as the second greatest vector of diseases in the world, after mosquitoes [6].

Of the diseases transmitted by ticks, rickettsiosis is one of the most important due to its severity [7]. The life cycle of rickettsiae involves vertebrate hosts and invertebrate vectors [8]. Ticks can acquire the bacteria through transovarian transmission (female to egg), which allows infected larvae to develop, maintaining and amplifying the infection in tick populations; by trans-stadial transmission (from the larva to the nymph and from the nymph to the adult); or by horizontal acquisition during feeding from a rickettsemic host [9,10,11]. 

In Latin America and the Caribbean, several *Rickettsia* species have been reported, which could be directly related to habitat fragmentation processes due to anthropogenic activities. This not only threatens the different ecosystems, but also alters the circulation of zoonotic agents, increasing the risk of infection in human populations [12]. 

Birds are important hosts in the life cycle of some tick species, mainly for the immature stages (larvae and nymphs). Some of these species are vectors of pathogens for animals and humans [13,14,15,16,17,18,19]. The role of birds in the spread of diseases has been documented since they can transport ticks and pathogens that are typical of certain areas to other distant regions during migrations [20,21], opening the possibility for a constant flow of ticks and pathogens [15,17,18,22,23,24,25]. 

Colombia has the largest number of bird species in the world, which have been studied due to their association with viral diseases such as avian influenza [26,27]. However, studies carried out by Londoño et al. [28], Cardona-Romero et al. [29] and Martínez-Sanchez et al. [30] suggest that birds have an important role in maintaining the circulation and spread of rickettsiae.

The objective of this study was to determine the species of ticks and rickettsiae in ticks associated with wild birds in an urban fragment of tropical dry forest in the north of Colombia. Additionally, monthly abundance patterns of ticks in vegetation were evaluated. Finally, given the abundance of ticks and *Rickettsia* in ticks collected from birds and of ticks collected from vegetation throughout the year, a meta-taxonomic analysis of 16S rRNA was performed in order to carry out a sensitive and rapid screening of Rickettsia and other bacteria both in bird blood samples and in ticks from the same area. 

## 2. Materials and Methods

### 2.1. Study Area

The study was carried out in an urban fragment of tropical dry forest with an area of 3 ha at the University of Magdalena (11°13′18.31″ N, 74°11′08.80″ W,) which is characterized by a semi-arid climate with a marked water deficit in the dry season. The precipitation regime is bimodal, with two periods of rain concentration, one from May to June and the other from September to November, with its greatest intensity in October, and two dry periods, the most intense being from December to April and a less intense one from July to August. The average monthly rainfall is 578 mm. The average annual temperature is 27 °C, the mean annual maximum temperature is 32.6 °C and the mean minimum is 23.3 °C [31,32].

### 2.2. Specimen Collection

The capture of birds was carried out using mist nets (12 × 2.5, 38 mm). Five mist nets were installed inside and on the edge of the forest, which were open from 6:00 a.m. to 12:00 p.m., with monitoring every 15 min for three days a week, during the months of January to April and August and September 2021.

For each captured specimen, the entire body was examined for ticks, which were collected from the distal part of the capitulum to avoid detachment of the hypostome using fine-tipped entomological tweezers and placed in labeled 1.5 mL Eppendorf tubes with absolute alcohol [16].

Blood samples were obtained from the brachial or jugular vein, taking 0.1 to 0.2 mL of blood per bird, taking into account the ratio of 0.06 mL of blood per 1 g of weight [33] using a syringe of 1 mL insulin and needles of 27G × ½. To avoid any risk of mortality, birds that weighed less than 10 g were not included for the blood sampling. All captured birds were identified with the help of the Ayerbe field guide [34], were ringed to determine recapture and were finally released.

To determine the seasonal variation in ticks in their free-living phase, monitoring was carried out every 15 days for a year, from February 2021 to February 2022, with a total of 24 samplings. For this, a dry forest trail was traversed, dragging a 1 × 1.30 m white fabric flag through the vegetation (dragging) for 90 min (starting at 8:00 am) and checking every 10 min [35]. These ticks were quantified, identified and stored in groups by species, stage and collection date in Eppendorf tubes with absolute alcohol at −20 °C. Additionally, from the temperature (T) and relative humidity (HR) records obtained from the Institute of Hydrology, Meteorology and Environmental Studies (IDEAM), the saturation deficit (DS) was calculated by applying the formula DS = (1 − HR/100) × 4.9463 × e(0.0621T) described by Randolph and Storey [36].

### 2.3. Morphological Identification of Ticks

Ticks were identified to the most specific level possible by following specific taxonomic keys and descriptions of Hooker et al. [37] and Martins et al. [38] and with the aid of a Zeiss Stemi 305 stereoscope and a Leica M205A motorized industrial microscope.

### 2.4. DNA Extractions and Amplifications

DNA extraction of ticks collected from birds was performed from the complete individual, individually or in groups of nymphs or larvae by species and by host, depending on the number of ticks found at each stage. A total of 7 nymphs and 235 larvae were extracted, obtaining 67 DNA samples (7 nymphs and 60 larval pools). Likewise, the DNA extraction of ticks collected in vegetation was grouped and processed in monthly pools and by stage. Extractions were performed using the Lucigen’s MasterPure™ Complete Kit.

To confirm the tick species, three pools of ticks were chosen at random for the amplification and sequencing of the cox1 Folmer et al. [39] and 16S Mangold et al. [40] genes (Supplementary Table S1). 

*Rickettsia* species from ticks collected from birds were identified by PCR (polymerase chain reaction) in an Eppendorf Mastercycler^®^ Pro thermal cycler, aimed at amplifying the gltA, sca1 and ompA genes [41,42] (Supplementary Table S1). 

DNA extractions from birds’ blood were performed on those individuals with positive ticks for *Rickettsia*. The extractions were performed individually using the E.N.Z.A Blood Omega kit. In all cases, DNA quality was confirmed by agarose gel electrophoresis and GelRed (Biotium) staining.

### 2.5. Analyses of Sequences

Positive PCR samples were sequenced in both directions. Sequences were edited on the BioEdit program [43] and were verified and compared with those deposited in GenBank using the NCBI BLAST tool (www.ncbi.nlm.nih.gov, accessed on 5 February 2022).

Additionally, the sequences obtained in this work, together with sequences available in GenBank, were further corroborated by phylogenetic methods using maximum likelihood (ML), building phylogenetic trees for each of the genes and then a tree with all the genes concatenated. The Geneious program [44] was used for the alignment of the sequences using the MAFFT algorithm [45], all the coding gene sequences (cox1, gltA, ompA and Sca1) were aligned by codons and the reading frame was corrected with the help of the AliView program [46] to eliminate the stop codons and subsequently cleaned in TranslatorX [47]. The sequences of the non-coding genes (16SrRNA) were aligned by nucleotides and subsequently cleaned with Gblocks 0.91b [48]. A partitioned concatenated file was constructed with the Geneious program [43] based on the models obtained by gen. IQ-TREE was used [49] for the selection of the best substitution models and for the phylogenetic analysis. Finally, FigTree V1.4.4 [50] was used to analyze and edit the generated phylogenetic trees. ML analyses were performed using the fast-scaling algorithm and 10,000 bootstrap pseudoreplicates (BPs). Bootstrap values >70% were considered to indicate high statistical support [51].

The best substitution models obtained were TN + F + I for codon positions 1, 2 and HKY + F + G4 for position 3 of the cox1 gene and TPM3 + F + G4 for positions 1, 2 and 3 of the gene 16S. Likewise, the best substitution models for codon positions 1, 2 and 3 of the gltA gene was K3PU + F + I, for ompA it was TPM3 + F + I and for Sca1 it was TPM3 + F + G4.

### 2.6. 16S Metataxonomy for the Identification of Rickettsiae in the Blood of Birds and Ticks Collected from Vegetation

A total of 23 blood DNA samples were grouped into 11 pools, by bird species or individually for bird species that only had one individual. We only worked with the blood samples of birds that presented ticks positive for rickettsia. 

A total of 233 ticks (208 larvae, 21 nymphs and 4 adults) collected in vegetation were grouped in 27 pools, by month and by stage. All DNA samples from blood and tick pools were quantified with a Nanodrop Multiskan go (Thermo scientific) for purity (260/280) > 1.6–2.2 and quality (260/230) > 1.8–2.2 parameters. We only worked with the DNA that met these parameters. In total, 38 pools were processed plus the positive control (ZymoBiomicsTM Microbial community DNA Standard—Zymo research) and negative controls (Supplementary Table S2).

For the preparation of the libraries, directed to the amplification of the V4 region of the 16S rRNA gene, the primers F515 and R806 were used [52]. The PCR reaction mixture was prepared in a volume of 25 µL, 2.5 µL of DNA ~ 5 ng/µL, 12.5 µL of Accuris High Fidelity Hot Start Master Mix 2X and 5 µL of each primer. DNA quality was quantified with a Qubit^®^ fluorometer (Life Technologies Carlsbad, Carlsbad, CA, USA) using a Qubit dsDNA HS^®^ Assay kit (Thermo Fisher Scientific Corporation, Waltham, MA, USA).

The resulting purified products were bound to dual indices using the Nextera XT IDX V2 kit in a reaction mix containing 2.5 µL of DNA, 2.5 µL each of Nextera index primers, 12.5 µL of 2x Accuris High Fidelity Hot Start Master Mix 2X and 5 µL of molecular-grade water. The final PCR product was quantified using a Qubit. The samples were normalized to calculate the volume to be taken from each sample for the multiplexed library, and a negative control sample was included as well as the PhiX Control v3 at 1 nM. Then, 1 nM of the final library was sequenced by paired-ended v.2 chemical sequencing (150 bp reads) along with their multiplex sample adapters on the Illumina iSeq100 platform (Illumina Inc., San Diego, CA, USA) according to the standard protocol (https://support.illumina.com/documents/documentation/chemistry_documentation/16s/16s-metagenomic-library-prep-guide-15044223-b.pdf, accessed on 10 June 2022) at the University of Magdalena.

### 2.7. Bioinformatic Analyses

The quality of the forward and reverse reads was assessed with fastp [53]. QIIME2 [54] was used to ensemble the metabarcoding data. First, the forward reads were imported and demultiplexed. Then, Amplicon Sequence Variants (ASVs) were generated with the DADA2 plugin [55]. Using the microDecon package with default settings [56], the samples were decontaminated using a negative control sample (blank) as a reference. To assess diversity, a reference-based phylogenetic tree using SEPP was produced with the q2-frament-insertion plugin [57]. Taxonomic assignment was performed with the qiime feature-classifier classify-sklearn plugin [58], using a classifier trained on the V4 region of the 16S rRNA from the SILVA database [59]. 

Downstream analysis was performed in R (version 4.1.2), using the phyloseq, animalcules, ampvis and microviz packages [60,61,62,63].

## 3. Results

### 3.1. Ticks Collected on Birds

A total of 367 birds were captured, distributed among 41 species, 35 genera, 17 families and 7 orders, of which 60 (16.35%) were found infested by ticks (524 larvae and 9 nymphs). The detail of the captured birds and the average abundance of ticks observed for each specie is presented in (Table 1).

All ticks were taxonomically identified as *Amblyomma* sp. or *Amblyomma dissimile*. Three pools were molecularly confirmed with Blast, obtaining identity percentages of 99.61%, 99.81% (MF095086.1) and 100% (MF095084.1) for cox1 and 99.47%, 100% (MF026013.1) and 100% (KY389392.1) for 16S with *A. dissimile* sequences available in GenBank.

This was confirmed by phylogenetic analyses by grouping our *A*. *dissimile* sequences (DL19, DL54 and DL89) with available *A. dissimile* sequences and the formation of a clade with *Amblyomma scutatum* and *Amblyomma rotundatum* sequences obtained from GenBank data, these being the most closely related species, with 100% bootstrap support (Supplementary Figure S1).

### 3.2. Rickettsiae in Ticks Collected from Birds

Of 60 larval and 7 nymphal pools, rickettsia DNA was detected in 47/60 (78%) larval pools and 7/7 (100%) nymphal pools. Of the 60 birds infested with ticks, 40 had ticks DNA positive for *Rickettsia*. The genera that presented the highest number of ticks with rickettsia infection were *Parkesia, Saltator*, *Campylorhynchus* and *Troglodytes*. Eleven species of birds were residents and two were migratory species (Table 2).

Sequences of the gltA, sca1 and ompA genes showed 100% identity with sequences of “*Candidatus* Rickettsia colombianensi” (MG563768.1; MN058024.1 and MG020421.1) or *Rickettsia* spp. (MH936461.1; MG020421.1) (Table 2). Phylogenies analyses confirmed that 100% of the rickettsia sequences obtained in this study correspond to “*Candidatus* Rickettsia colombianensi” (Supplementary Figure S2).

### 3.3. Ticks Collected from Vegetation and Seasonality

**Figure 1:**
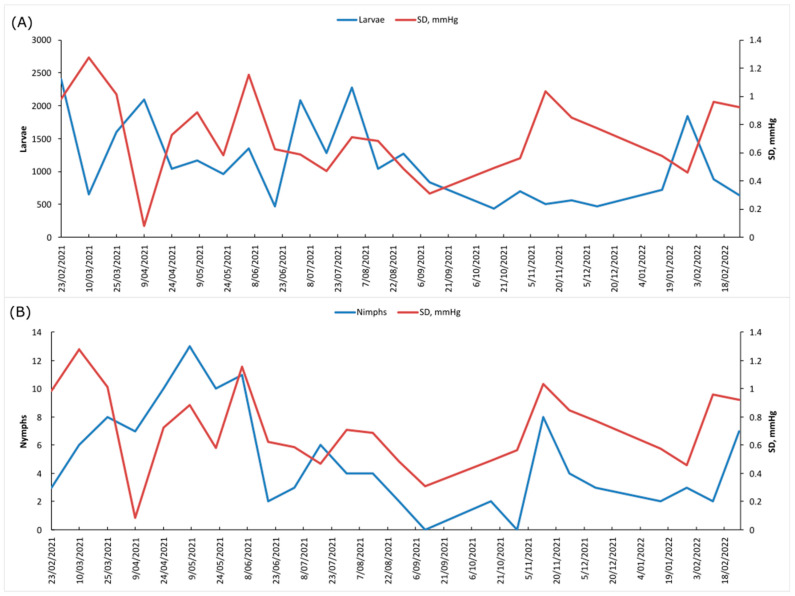
Saturation deficit (SD mmHg) of the tropical dry forest in relation to the abundance of ticks collected between February 2021 and February 2022 for (**A**) larvae, (**B**) nymphs.

A total of 27,405 ticks were captured on vegetation, of which only 121 were nymphs and 5 were adults. All were identified as *Amblyomma* sp. or *Amblyomma dissimile*. In general, the largest number of larvae and nymphs was captured between the months of March and July, while adults were captured in January and February. A fluctuation between the abundance of larvae per sampling day could be observed in relation to the saturation deficit (DS) (Figure 1A); however, this did not affect the abundance of nymphs (Figure 1B).

### 3.4. 16S amplicon Sequence Analyses of Blood Samples and Ticks from Vegetation

For the 12 samples of blood from birds infested with ticks and the 27 samples of ticks collected from vegetation, a total of 3,438,420 paired-end reads were obtained. A negative control and a Mock community were also sequenced to evaluate contamination and composition bias. Quality assessment showed an even number of reads for all experimental samples (Supplementary Figure S3). As a result of the low quality at the end of the reverse reads, no overlap could be computed. Thus, a single-end approach using the forward reads was used. 

**Figure 2:**
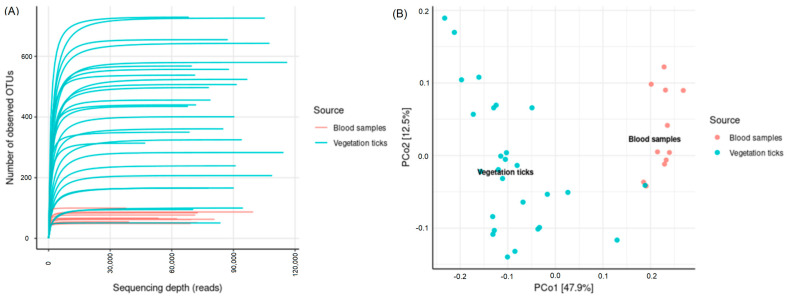
(**A**) Rarefaction curves showing the number of observed ASVs for all sequenced samples, highlighting type of origin of each sample: blood from birds and ticks collected from vegetation. (**B**) Principal Coordinate Analysis (PCoA) based on weighted UniFrac distances for all samples. The source of the sample (blood sample and ticks collected from vegetation) in shown in different colors.

A total of 6897 ASVs were generated and assigned to a taxonomic rank. Rarefaction curves were generated to evaluate if the depth of sequencing was sufficient to capture all diversity in the samples. Figure 2A shows that the number of observed ASVs for all samples levels off before sequencing depth is lost. There are notable differences between blood and vegetation samples in terms of the number of ASVs observed. The number of ASVs from blood samples is not as big as for ticks collected from vegetation, and the blood samples’ display less spread. 

Principal Coordinate Analysis (PCoA) showed that most of the variation (PC1, 47.9%) was explained by the source of the sample, i.e., blood of birds or ticks from vegetation (Figure 2B). Most of the samples group closely, except for a sample from a nymph collected from vegetation in the month of December, which clustered with the blood samples. The second principal component (PC2, 12.5%) displayed the variation between the ticks collected from vegetation, which may be explained by the seasonality of the sampling and the different stage of the ticks.

**Figure 3:**
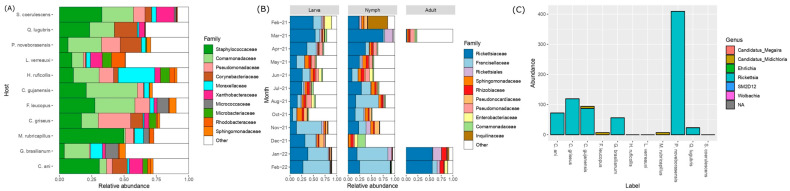
Taxonomic composition on the relative abundance for (**A**) the bird blood samples according to their host, (**B**) the vegetation samples for each month, with each life cycle stage separated. (**C**) Total abundance of the Rickettsiales Order for the blood birds’ samples, distinguishing between different genera present.

The taxonomic composition of the samples at the Family level was compared between each species of bird sampled for blood, and for ticks collected from vegetation, the month when they were captured, separating for the ticks’ life stage (Figure 3A). Rickettsiaceae was the most abundant family for the vegetation samples, followed by Francisellaceae and the Order Rickettsiales. This last taxon comprises AVSs whose taxonomic classification could not be placed higher than Order rank. January, February and March were the months with the most relative abundance of Rickettsiaceae, but it was found throughout the year. 

For the bird samples, the taxa with the most abundance were Staphylococcaceae, Comamonadaceae and Pseudomonadaceae (Figure 3B). *Rickettsia* spp. was present in low abundance in bird samples, but it was clearly detected in the species *P. noveborasensis*, *C. griseus*, *C. gujanensis*, *C. ani* and *G. brasilianum* (Figure 3C). Shannon index alpha diversity analysis showed no significant differences within samples (Kruskal–Wallis rank sum test, *p* < 0.05). The PERMANOVA test using Bray distance showed a significant beta diversity difference between ticks collected from vegetation and blood samples (*p* < 0.05) (Supplementary Figure S4).

## 4. Discussion

This study reports the parasitism of *Amblyomma dissimile* on wild birds in an urban fragment of tropical dry forest in the department of Magdalena. *A. dissimile* ticks were also found in great number and throughout the year in the vegetation from the same area. *A. dissimile* is a tick with a large number of hosts [64]. In Colombia, it has been found parasitizing mammals, such as *Hydrochoerus hydrochaeris* and *Proechimys semispinosus*; amphibians such as *Rhinella humboldti* and *Rhinella horribilis*; reptiles such as *Iguana iguana*; domestic animals such as canines, cattle and horses; as well as humans [28,40,65,66,67,68,69,70,71,72].

As in our study, Cotes-Perdomo et al. [71,73] and Santodomingo et al. [40] reported *A. dissimile* in ticks present in the tropical dry forest in the departments of Cesar, La Guajira and Magdalena, Colombia. However, the tick stages found in their study were adults and nymphs, unlike our study, where the vast majority were larvae and a few nymphs, which could be related to the habits of the host birds. The bird species reported with the highest number of ticks (*Campylorhynchus griseus*, *Furnarius leucopus*, *Parkesia noveboracensis* and *Troglodytes aedon*) usually wander in the leaf litter and/or low vegetation [34], where the larvae and some nymphs are usually found; after molting, they hydrate in the plant layer, a few millimeters above the ground [74].

Of the 18 species of birds collected with ticks, 13 species had positive ticks for *Rickettsia*, and from those, 11 species are residents of the dry forest, i.e., *Troglodytes aedon*, *Leptotila verreauxi*, *Saltator coerulescens*, *Cyclarhis gujanensis*, *Campylorhynchus griseus*, *Hypnelus ruficollis*, Dendroplex picus, *Furnarius leucopus*, *Glaucidium brasilianum*, *Quiscalus lugubris* and *Melanerpes rubricapillus*, and two are migratory species, i.e., *Catharus minimus* and *Parkesia noveborasensis* (Table 2). This interaction between *A. dissimile* and the mentioned bird species had not been previously reported. However, in Toronto, Canada, the finding of *A. dissimile* in the species *Catharus fuscescens* was reported, and the authors argued that probably it could have acquired the tick during its migratory flight [20]. This species belongs to the same genus as the migratory species *Catharus minimus* reported in our study with an *A. dissimile* tick positive for “*Candidatus* Rickettsia colombianensi” infection. Both species migrate to spend the winter in Central and South America [34].

In Colombia, nine species of ticks have been reported parasitizing wild birds: *A. dissimile*, *Amblyomma longirostre*, *Amblyomma ovale*, *Amblyomma varium*, *Amblyomma nodosum*, *Amblyomma calcaratum*, *Amblyomma mixtum*, *Haemaphysalis leporispalustris* and *Ixodes* sp. [29,30,75]. However, only two studies report Rickettsia infestation in ticks collected from wild birds, one of which is that of Martínez-Sanchez et al. [30], who reported the parasitism of “*Candidatus* Rickettsia colombianensi” in *A. dissimile* collected in the boreal migratory bird species *Oporornis agilis* in the department of Caldas. Likewise, this author also reported *Rickettsia amblyommatis* in *Amblyomma longirostre*, *Amblyomma varium* and *Ixodes* sp., obtaining a general prevalence of 11%, lower than that obtained in our study. Cardona-Romero et al. [29] reported Rickettsia parkeri in Amblyomma nodosum in Arauca, and, as in our study, the bird species T. aedon was the one that showed the greatest infestation by ticks.

No significant difference was observed in the abundance of ticks collected from birds during the months sampled. However, when comparing the mean abundance of ticks per family of bird, we found that in our study, it was higher than that obtained in the studies carried out by Cardona-Romero et al. [29] and Martínez-Sanchez et al. [76], also in Colombia (Table 1). We believe that this is due to the characteristics of the study area, as they sampled areas with abundant levels of precipitation throughout the year and floodplain ecosystems. Although ticks are cosmopolitan, most are found mainly in warm climates [4,77]. It is worth mentioning that the greater interactions observed between ticks and rickettsia in Cardona-Romero et al. [29] and Martínez-Sanchez et al. [76] studies may be due to the fact that these were carried out in larger study areas, which included 8 to 11 localities, including elevations from 178 to 3845 m. Our study was carried out in an urban fragment of dry forest, surrounded by an urban matrix.

The results of this study suggest that *A. dissimile* did not show a clear seasonal pattern, since its stages were found in similar proportions throughout the year, except for adults. This result implies that it would be difficult to identify in which periods of the year people or animals would be at greater risk of acquiring ticks or tick pathogens. Unlike those ticks that inhabit temperate climates or subpolar regions that have marked seasonal periods, in synchroneity with the seasons of the year [78].

In general, the largest number of larvae and nymphs were collected between the months of March and July, while adults were captured in January and February. This coincides with the values of the saturation deficit (SD) (Figure 1), since high percentages of relative humidity (RH) and low average temperature (T) cause the SD to decrease. It should be noted that SD did not affect the abundance of nymphs, since it has been shown that low percentages of HR, low average T and high SD are detrimental during the incubation period of tick eggs and for the larval stages that are more sensitive to desiccation [35]. This pattern was also reflected in a slight relationship in the months with the highest abundance of ticks on birds and vegetation.

Given the abundance of *Rickettsia* in ticks collected from birds and of ticks collected from vegetation throughout the year, a meta-taxonomic analysis of 16S rRNA was performed to screen for *Rickettsia* and other bacteria both in blood samples from birds and in ticks from the same area. The metataxonomic analysis of 16S detected the presence of rickettsiae in all the samples of ticks collected in the vegetation in high abundance. Considering that 99% of the ticks collected were larvae, we could suggest the transovarial route of transmission of rickettsia as the main mechanism present, also reported as an amplifying mechanism in ticks [11]. Unlike our study, Kantsø et al. [79] reported in Denmark the presence of *Rickettsia helvetica* by amplifying the gltA gene in 31/662 *Ixodes ricinus* nymphs, while none of the larvae carried Rickettsial DNA, the highest rate of ticks positive for *R. helvetica* was in the month of May. These months are hotter, humid and sunny, conditions that favor fitness, rates of interstadial development and behavior of most tick species [78,80,81,82]. These conditions are present most of the year in tropical countries, which, in our study, correlates with the SD and would explain the presence of *Rickettsia* in ticks collected throughout the year. Another associated factor is the high level of fragmentation of the study area, which also alters the natural circulation of pathogens in ecosystems [12].

Due to the location of the study area, within an urban matrix, our result should be of importance from a public health perspective. As Quintero et al. [83] points out, the detection of *Rickettsia* in ticks that are potential ectoparasites to humans, as is the case of *A. dissimile*, should be of concern, as the pathogenic potential of *Rickettsia* such as “*Candidatus* Rickettsia colombianensi” has not been discarded.

*Rickettsia* was also detected in low abundance in bird blood samples. To our knowledge, this is the first study aiming to detect *Rickettsia* in blood of birds using 16S rRNA metabarcoding. Similar studies by Stanczak et al. [84] in Poland for the detection of *Rickettsia* in birds, deer and rodents using PCR did not show positive results in bird blood samples; however, studies such as the one by Ioannou et al. [85] reported 1.5% of bird blood samples positive by PCR for an unknown rickettsia species. Likewise, Spitalska et al. [86] reported the infection of rickettsia in blood in the bird species *Periparus ater*. Hornok et al. [87] also reported 4.7% of their bird blood samples positive for *R. helvetica*; however, these authors reported two interesting cases, one in which only 2 out of 11 ticks removed from a PCR-positive bird were PCR-positive for *R. helvetica*, and a second case in which a bird positive for *R. helvetica* had only one tick with a negative PCR test. This may suggest that rickettsemia may persist after tick vector shedding in relevant birds and that transmission of rickettsiae from birds to tick vectors may occur inefficiently, especially if the host is not suitable.

However, these authors suggest that the role of vertebrates as reservoirs in the natural life cycle of rickettsiae, capable of transmitting and infecting ticks, remains under debate and study [85,86,87,88,89,90,91], especially when the pathogen is transmitted transovarially, as is the case with many *Rickettsia* species [92] and when vertebrates are rickettsiaemic only for short periods of time, since they are unable to maintain rickettsemia long enough to infect new uninfected ticks [93,94,95]. It is important to bear in mind that in vertebrates, the endothelial tissues are the main target of some species of *Rickettsia*—it is in the tissues where, after the bite of the vector, the bacteria lodge and find the necessary physiological conditions to infect the cells and multiply [96,97]. 

## 5. Conclusions

This study reports for the first time the presence of “*Candicatus* Rickettsia colombianensi” in *Ambyomma dissimile* ticks collected from wild birds in an urban fragment of tropical dry forest in Santa Marta, Colombia. Some species of birds showed a high mean abundance of tick parasitism compared to other studies. The seasonality of larvae and nymphs suggest that they are active throughout the year. The usefulness of the 16S meta-taxonomy technique to quickly and cost-effectively assess the presence of Rickettsiae or other bacteria in ticks and birds’ blood was confirmed.

## Figures and Tables

**Table 1:** Order, Family, species and quantity of captured birds infested by *Amblyomma dissimile.* Includes information on the tick stages collected in each bird species and mean abundance (MA) of ticks from each stage per bird species. * Migratory bird.

Birds				Ticks			
Order	Family	Species	No. of Birds Infested/No. of Birds Captured (%)	Larva	Nymph	Mean Abundance	
						Larva	Nymph
Passeriformes	Passerellidae	*Arremonops conirostris*	0/2 (0)				
	Troglodytidae	*Campylorhynchus griseus*	6/6 (100)	223		37.2	
		*Troglodytes aedon*	5/9 (55)	118	5	13.1	0.6
	Tyrannidae	*Contopus virens*	0/8 (0)				
		*Megarynchus pitangua*	0/1 (0)				
		*Pitangus sulphuratus*	2/10 (20)	6		0.6	
		*Myiarchus Tyrannulus*	0/4 (0)				
		*Myiodynastes maculatus*	0/2 (0)				
		*Tyrannus melancholicus*	0/11 (0)				
	Parulidae	*Geothlypis philadelphia*	0/1 (0)				
		Parkesia noveboracensis *	10/15 (66)	51		3.4	
		*Protonotaria citrea*	0/4 (0)				
		*Setophaga petechia*	0/23 (0)				
	Cardinalidae	*Pheucticus ludovicianus*	0/2 (0)				
		*Piranga rubra*	0/2 (0)				
	Furnariidae	*Dendroplex picus*	1/1 (100)	2		2.0	
		*Furnarius leucopus*	4/8 (50)	19		2.4	
	Icteridae	*Icterus nigrogularis*	1/40 (2)	1		0.0	
		*Quiscalus lugubris*	5/22 (22)	10		0.5	
		*Quiscalus Mexicanus*	0/2 (0)				
	Thraupidae	*Saltator coerulescens*	8/49 (16)	18		0.4	
		*Volatinia jacarina*	0/10 (0)				
		*Thraupis episcopus*	0/7 (0)				
	Turdidae	Catharus minimus *	1/4 (25)	3		0.8	
		*Turdus leucomelas*	1/2 (50)	5		2.5	
	Mimidae	*Mimus gilvus*	0/1 (0)				
	Vireonidae	*Cyclarhis gujanensis*	3/7 (42)	3		0.4	
Columbiformes	Columbidae	*Leptotila verreauxi*	3/17 (17)	9		0.5	
		*Columbina passerina*	0/4 (0)				
		*Columbina squammata*	1/8 (12)	10		1.3	
		*Columbina talpacoti*	0/14 (0)				
Cuculiformes	Cuculidae	Coccyzos americanus *	1/2 (50)	2		1.0	
		*Crotophaga ani*	2/8 (25)	1	1	0.1	0.1
		*Crotophaga major*	0/1 (0)				
Piciformes	Picidae	*Melanerpes rubricapillus*	2/22 (9)	2		0.0	
		*Colaptes punctigula*	0/1 (0)				
Psittaciformes	Psittacidae	*Eupsittula pertinax*	0/2 (0)				
Strigiformes	Strigidae	*Glaucidium brasilianum*	1/2 (50)	18		9.0	
Galbuliformes	Bucconidae	*Hypnelus ruficollis*	3/3 (100)	23	3	7.7	1.0
		Total:		524	9		

**Table 2:** Species of birds with ticks infested by *Rickettsia*. L = Larva, N = Nymph, * migratory bird.

Species of Bird Host	(# of Ticks/Stage)	No. Birds with Ticks Infested by Rickettsia/No. Birds with Ticks Tested (%)	Blast Best Identity Match
*Hypnelus ruficollis*	(23/L; 3/N)	3/3 (100)	“*Candidatus* R. colombianensi” (MN058024.1) 100%
*Campylorhynchus griseus*	(223/L)	4/6 (66)	“*Candidatus* R. colombianensi” (MF428453.1) 100% and *Rickettsia* sp. (MG020421.1) 99.82%
*Troglodytes aedon*	(118/L; 5/N)	5/5 (100)	“*Candidatus* R. colombianensi” (MN058024.1) 100% and *Rickettsia* sp. (MG020421.1) 100%
*Leptotila verreauxi*	(9/L)	3/3 (100)	“*Candidatus* R. colombianensi” (MN058024.1) 99.55% and *Rickettsia* sp. (MG020421.1) 100%
*Saltator coerulescens*	(18/L)	4/8 (50)	“*Candidatus* R. colombianensi” (MN058024.1) 100% and *Rickettsia* sp. (MG020421.1) 99.83%
*Cyclarhis gujanensis*	(3/L)	2/3 (66)	“Candidatus R. colombianensi” (MG563768.1) 100% and *Rickettsia* sp. (MG020421.1) 100%
*Melanerpes rubricapillus*	(2/L)	1/2 (50)	*Rickettsia* sp. (MG020420.1) 100%
*Icterus nigrogularis*	(1/L)	0/1 (0)	
*Furnarius leucopus*	(19/L)	3/4 (74)	*Rickettsia* sp. (MH936461.1) 100%
*Columbina squammata*	(10/L)	0/1 (0)	
*Crotophaga ani*	(1/L; 1/N)	1/2 (50)	
*Dendroplex picus*	(2/L)	1/1 (100)	“*Candidatus* R. colombianensi” (MN058024.1) 99.73% and *Rickettsia* sp. (MG020421.1) 99.65%
*Turdus leucomelas*	(5/L)	0/1 (0)	
*Pitangus sulphuratus*	(6/L)	0/2 (0)	
Catharus minimus *	(3/L)	1/1 (100)	“*Candidatus* R. colombianensi” (MN058024.1) 100% and *Rickettsia* sp. (MG020421.1) 100%
Coccyzos americanus *	(2/L)	0/1 (0)	
*Glaucidium brasilianum*	(18/L)	1/1 (100)	*Rickettsia* sp. (MH936461.1) 100%
*Quiscalus lugubris*	(10/L)	2/5 (40)	“*Candidatus* R. colombianensi” (MN058024.1) 99.45 and *Rickettsia* sp. (MG020421.1) 100%
Parkesia noveborasensis *	(51/L)	9/10 (90)	“*Candidatus* R. colombianens*i”* (MN058024.1) 100% and *Rickettsia* sp. (MG020421.1) 100%
	Total:	40/60 (66%)	

## Data Availability

All the sequences generated in this study were published in GenBank under accession numbers (ON638923-ON638951) gltA, (ON664949-ON664973) ompA, (ON664974-ON665005) sca1, (OM912709-OM912711) cox1 and (OM943426-OM943428) 16S. All raw sequencing data from the 16S meta-taxonomic analysis have been deposited in the NCBI Sequence Read Archive (SRA) under BioProject ID PRJNA914061.

## Supplementary Materials

The following supporting information can be downloaded at: https://www.mdpi.com/article/10.3390/life13010145/s1, Table S1: Primers used for the molecular identification of Ticks and Rickettsia; Table S2: Blood samples from birds and ticks from vegetation processed by 16S metataxonomy amplicon sequencing; Figure S1: Phylogenetic reconstruction using maximum likelihood of the concatenated CO1 and 16SrDNA Amblyomma sequences obtained in this study and others downloaded from GenBank (with accession numbers included cox1/16S). Numbers on nodes correspond to bootstrap values; Figure S2: Phylogenetic reconstruction using maximum likelihood of concatenated gltA, ompA and Sca1 genes for Ricketssia, including our sequences and sequences downloaded from GenBank (with accession numbers included gltA/ompA/Sca1). Numbers on nodes correspond to bootstrap values; Figure S3: Number of reads for all samples. For the bird blood samples, the scientific name of the host where it was isolated is used. For tick samples collected from vegetation, the number represents the month of collection (1–12 for all samples taken in 2021, 22 for the February 2022 samples), the letter represents the ticks’ life stage: (L)arva, (N)ymph, (A)dult; Figure S4: Alpha diversity analysis based on Shannon index showed no significant differences (Kruskal-Wallis’s rank sum test, *p* < 0.05) between (A) Ticks life stage and (B) vegetation ticks and blood samples. PERMANOVA test using Bray distance did showed significant beta diversity difference existing between vegetation ticks and blood samples (*p* < 0.05).

## References

[1] Dabanch J. (2003). Zoonosis. Rev. Chilena. Infectol..

[2] Szyfres B. (2003). Zoonosis y Enfermedades Transmisibles Comunes al Hombre y a los Animales.

[3] Guglielmone A., Estrada-Peña A., Keirans E., Robbins G. (2003). Ticks (Acari: Ixodidae) of the neotropical zoogeographic region. Ticks Tick Borne Dis..

[4] Guglielmone A., Bechara H., Szabó P., Barros M., Faccini L., Labruna M., De La Vega R., Arzua M., Campos M., Furlong J. (2004). Garrapatas de importancia médica y veterinaria: América Latina y El Caribe. Ticks Tick Borne Dis..

[5] Horak G., Camicas L., Keirans E. (2002). The Argasidae, Ixodidae and Nuttalliellidae (Acari: Ixodida): A world list of valid tick names. Exp. Appl. Acarol..

[6] Jongejan F., Uilenberg G. (2004). The global importance of ticks. Parasitology.

[7] Ortiz J., Miranda J., Ortiz L., Navarro Y., Mattar S. (2015). Seroprevalencia de *Rickettsia* sp. en indígenas Wayuü de la Guajira y Kankuamos del Cesar, Colombia. Infectio.

[8] Vélez J.C.Q., Hidalgo M., González J.D.R. (2012). Rickettsiosis, una enfermedad letal emergente y re-emergente en Colombia. Univ. Sci..

[9] Raoult D., Dutour O., Houhamdi L., Jankauskas R., Fournier P.E., Ardagna Y., Aboudharam G. (2006). Evidence of louse-borne diseases in soldiers of Napoleon’s Grand Army in Vilnius. J. Infect. Dis..

[10] Walker D.H., Ismail N. (2008). Emerging and re-emerging rickettsioses: Endothelial cell infection and early disease events. Nat. Rev. Microbiol..

[11] Ramírez-Hernández A. (2014). Identificación molecular y análisis de la relación filogenética de especies de Rickettsias presentes en garrapatas provenientes de tres regiones de Colombia. Mate’s Thesis.

[12] Ogrzewalska M., Uezu A., Jenkins C.N., Labruna M.B. (2011). Effect of forest fragmentation on tick infestations of birds and tick infection rates by Rickettsia in the Atlantic Forest of Brazil. EcoHealth.

[13] Ogrzewalska M., Pacheco R.C., Uezu A., Richtzenhain L.J., Ferreira F., Labruna M.B. (2009). Ticks (Acari: Ixodidae) infesting birds in an atlantic rain forest region of Brazil. J. Med. Entomol..

[14] Sonenshine D.E., Lane R.S., Nicholson W.L. (2002). Chapter 27: Ticks (ixodida). Medical and Veterinary Entomology.

[15] Parola P., Paddock C.D., Socolovschi C., Labruna M.B., Mediannikov O., Kernif T., Abdad M.Y., Stenos J., Bitam I., Fournier P.E. (2013). Update on tick-borne rickettsioses around the world: A geographic approach. Clin. Microbiol. Rev..

[16] Flores F.S., Nava S., Batall´an G., Tauro L.B., Contigiani M.S., Diaz L.A., Guglielmone A.A. (2014). Ticks (Acari: Ixodidae) on wild birds in north-central Argentina. Ticks Tick Borne Dis..

[17] Ramos D.G.D.S., Melo A.L., Martins T.F., Alves A.D.S., Pacheco T.D.A., Pinto L.B., Pacheco R.C. (2015). Rickettsial infection in ticks from wild birds from Cerrado and the Pantanal region of Mato Grosso, midwestern Brazil. Ticks Tick Borne Dis..

[18] Luz H.R., Faccini J.L.H., McIntosh D. (2017). Molecular analyses reveal an abundant diversity of ticks and rickettsial agents associated with wild birds in two regions of primary Brazilian Atlantic Rainforest. Ticks Tick Borne Dis..

[19] Vivas R.I.R., Chi M.O., González M.B., Aguilar J.A.R. (2019). Las garrapatas como vectores de enfermedades zoonóticas en México. Bioagrociencias.

[20] Scott J.D., Durden A.L. (2015). First record of *Ambyomma rotundatum* tick (Acari: Ixodidae) parasitizing a bird collected in Canada. Syst. Appl. Acarol..

[21] Scott J.D., Durden L.A. (2015). *Amblyomma dissimile* Koch (Acari: Ixodidae) parasitizes bird captured in Canada. Syst. Appl. Acarol..

[22] Scott J., Fernando K., Banerjee S., Durden L., Byrne S., Banerjee M., Mann R., Morshed M. (2001). Birds Disperse Ixodid (Acari: Ixodidae) and Borrelia burgdorferi-Infected Ticks in Canada. J. Med. Entomol..

[23] Mukherjee N., Beati L., Sellers M., Burton L., Adamson S., Robbins R.G., Moore F., Karim S. (2014). Importation of exotic ticks and tick-borne spotted fever group rickettsiae into the United States by migrating songbirds. Ticks Tick Borne Dis..

[24] Cohen E.B., Auckland L.D., Marra P.P., Hamer S.A. (2015). Avian migrants facilitate invasions of neotropical ticks and tick-borne pathogens into the United States. Appl. Environ. Microbiol..

[25] Budachetri K., Williams J., Mukherjee N., Sellers M., Moore F., Karim S. (2017). The microbiome of neotropical ticks parasitizing on passerine migratory birds. Ticks Tick Borne Dis..

[26] Munster V.J., Fouchier R.A.M. (2009). Avian influenza virus: Of virus and bird ecology. Vaccine.

[27] Vélez D., Tamayo E., Ayerbe-Quiñones F., Torres J., Rey J., Castro-Moreno C., Ochoa-Quintero J.M. (2021). Distribution of birds in Colombia. BDJ.

[28] Londoño A.F., Díaz F.J., Valbuena G., Gazi M., Labruna M.B., Hidalgo M., Rodas J.D. (2014). Infection of *Amblyomma ovale* by *Rickettsia* sp. strain Atlantic rainforest, Colombia. Ticks Tick Borne Dis..

[29] Cardona-Romero M., Martínez-Sánchez E.T., Londono J.A., Tobón-Escobar W.D., Ossa-López P.A., Pérez-Cárdenas J.E., Rivera-Páez F.A. (2020). Rickettsia parkeri strain Atlantic rainforest in ticks (Acari: Ixodidae) of wild birds in Arauca, Orinoquia region of Colombia. Int. J. Parasitol. Parasites Wildl..

[30] Martínez-Sánchez E.T., Cardona-Romero M., Ortiz-Giraldo M., Tobón-Escobar W.D., Moreno-López D., Ossa-López P.A., Rivera-Páez F.A. (2021). *Rickettsia* spp. en garrapatas (Acari: Ixodidae) de aves silvestres en Caldas, Colombia. Acta Trop..

[31] Rangel-Ch O., Garzón A. (1995). Sierra Nevada de Santa Marta (Colombia). Colombia Diversidad Biótica.

[32] Rangel-Ch J.O. (2012). Colombia Diversidad Biótica XII: La región Caribe de Colombia.

[33] Hoysak D.J., Weatherhead P.J. (1991). Sampling blood from birds: A technique and an assessment of its effect. Condor.

[34] Ayerbe Quiñones F.A. (2019). Guía Ilustrada de la Avifauna Colombiana.

[35] Tarragona E.L., Sebastian P.S., Bottero M.N.S., Martinez E.I., Debárbora V.N., Mangold A.J., Nava S. (2018). Seasonal dynamics, geographical range size, hosts, genetic diversity and phylogeography of Amblyomma sculptum in Argentina. Ticks Tick Borne Dis..

[36] Randolph S.E., Storey K. (1999). Impact of microclimate on immature tick rodent host interactions (Acari: Ixodidae): Implications for parasite transmission. J. Med. Entomol..

[37] Hooker W.A., Bishopp F.C., Wood H.P. (1912). The Life History and Bionomics of some North American Ticks.

[38] Martins T.F., Onofrio V.C., Barros-Battesti D.M., Labruna M.B. (2010). Nymphs of the genus Amblyomma (Acari: Ixodidae) of Brazil: Descriptions, redescriptions, and identification key. Ticks Tick Borne Dis..

[39] Folmer O., Black M., Hoeh W., Lutz R., Vrijenhoek R. (1994). DNA primers for amplification of mitochondrial cytochrome c oxidase subunit I from diverse metazoan invertebrates. Mol. Mar. Biol. Biotechnol..

[40] Mangold A.J., Bargues M.D., Mas-Coma S. (1998). Mitochondrial 16S rDNA sequences and phylogenetic relationships of species of Rhipicephalus and other tick genera among Metastriata (Acari: Ixodidae). Parasitol. Res..

[41] Labruna M.B., Whitworth T., Bouyer D.H., McBride J., Camargo L.M.A., Camargo E.P., Walker D.H. (2004). Rickettsia bellii and Rickettsia amblyommii in Amblyomma ticks from the State of Rondonia, Western Amazon, Brazil. J. Med. Entomol..

[42] Santodomingo A., Cotes-Perdomo A., Foley J., Castro L.R. (2018). Rickettsial infection in ticks (Acari: Ixodidae) from reptiles in the Colombian Caribbean. Ticks Tick Borne Dis..

[43] Hall T., Biociencias I., Carlsbad C. (2011). BioEdit: Un software importante para la biología molecular. GERF Bull. Biosci..

[44] Kearse M., Moir R., Wilson A., Stones-Havas S., Cheung M., Sturrock S., Drummond A. (2012). Geneious Basic: An integrated and extendable desktop software platform for the organization and analysis of sequence data. Bioinformatics.

[45] Katoh K., Toh H. (2008). Recent developments in the MAFFT multiple sequence alignment program. Brief Bioinform..

[46] Larsson A. (2014). AliView: A fast and lightweight alignment viewer and editor for large datasets. Bioinformatics.

[47] Abascal F., Zardoya R., Telford M.J. (2010). TranslatorX: Multiple alignment of nucleotide sequences guided by amino acid translations. Nucleic Acids Res..

[48] Castresana J. Gblocks, v. 0.91 b. http://molevol.cmima.csic.es/castresana.Gblocks_server.Html.

[49] Trifinopoulos J., Nguyen L.T., von Haeseler A., Minh B.Q. (2016). W-IQ-TREE: A fast online phylogenetic tool for maximum likelihood analysis. Nucleic Acids Res..

[50] Rambaut A. (2009). FigTree. Tree Figure Drawing Tool. http://tree.bio.ed.ac.uk/software/figtree/.

[51] Hillis D.M., Bull J.J. (1993). An empirical test of bootstrapping as a method for assessing confidence in phylogenetic analysis. Syst. Biol..

[52] Caporaso J.G., Lauber C.L., Walters W.A., Berg-Lyons D., Lozupone C.A., Turnbaugh P.J., Knight R. (2011). Global patterns of 16S diversity at a depth of millions of sequences per sample. Proc. Natl. Acad. Sci. USA.

[53] Chen S., Zhou Y., Chen Y., Gu J. (2018). fastp: An ultra-fast all-in-one FASTQ preprocessor. Bioinformatics.

[54] Bolyen E., Rideout J.R., Dillon M.R. (2019). Reproducible, interactive, scalable and extensible microbiome data science using QIIME 2. Nat. Biotechnol..

[55] Callahan B.J., Paul J.M., Michael J.R., Andrew W.H., Amy Jo A.J., Susan P.H. (2016). DADA2: High-resolution sample inference from Illumina amplicon data. Nat. Methods.

[56] McKnight D.T., Roger H., Deborah S.B., Lin S., Ross A.A., Kyall R.Z. (2019). microDecon: A highly accurate read-subtraction tool for the post-sequencing removal of contamination in metabarcoding studies. Environ. DNA.

[57] Janssen S., McDonald D., Gonzalez A., Navas-Molina J.A., Jiang L., Xu Z.Z., Winker K., Kado D.M., Orwoll E., Manary M. (2018). Phylogenetic placement of exact amplicon sequences improves associations with clinical information. Msystems.

[58] Bokulich N.A., Kaehler B.D., Rideout J.R. (2018). Optimizing taxonomic classification of marker-gene amplicon sequences with QIIME 2’s q2-feature-classifier plugin. Microbiome.

[59] Quast C., Pruesse E., Yilmaz P., Gerken J., Schweer T., Yarza P., Peplies J., Glöckner F.O. (2012). The SILVA ribosomal RNA gene database project: Improved data processing and web-based tools. Nucleic Acids Res..

[60] McMurdie P.J., Holmes S. (2013). phyloseq: An R package for reproducible interactive analysis and graphics of microbiome census data. PloS ONE.

[61] Andersen K.S., Kirkegaard R.H., Karst S.M., Albertsen M. (2018). ampvis2: An R package to analyse and visualise 16S rRNA amplicon data. BioRxiv.

[62] Barnett D.J., Arts I.C., Penders J. (2021). microViz: An R package for microbiome data visualization and statistics. J. Open Source Softw..

[63] Zhao Y., Federico A., Faits T., Manimaran S., Segrè D., Monti S., Johnson W.E. (2021). animalcules: Interactive microbiome analytics and visualization in R. Microbiome.

[64] Nava S., Venzal J.M., Acuña D.G., Martins T.F., Guglielmone A.A. (2017). Ticks of the Southern Cone of America: Diagnosis, Distribution, and Hosts with Taxonomy, Ecology and Sanitary Importance.

[65] Miranda J., Portillo A., Oteo J.A., Mattar S. (2012). *Rickettsia* sp. strain colombianensi (Rickettsiales: Rickettsiaceae): A new proposed Rickettsia detected in Amblyomma dissimile (Acari: Ixodidae) from iguanas and free-living larvae ticks from vegetation. J. Med. Entomol..

[66] Miranda J., Mattar S. (2014). Molecular detection of Rickettsia bellii and *Rickettsia* sp. strain Colombianensi in ticks from Cordoba, Colombia. Ticks Tick Borne Dis..

[67] Quintero J.C., Londoño A.F., Díaz F.J., Agudelo-Flórez P., Arboleda M., Rodas J.D. (2013). Ecoepidemiología de la infección por rickettsias en roedores, ectoparásitos y humanos en el noroeste de Antioquia, Colombia. Biomédica.

[68] Quintero V.J.C., Paternina T.L.E., Uribe Y.A., Muskus C., Hidalgo M., Gil J., Rojas A.C. (2017). Análisis ecoepidemiológico de la seropositividad rickettsial en zonas rurales de Colombia: Un enfoque multinivel. PLoS ONE.

[69] Rivera-Páez F.A., Labruna M.B., Martins T.F., Perez J.E., Castaño-Villa G.J., Ossa-López P.A., Camargo-Mathias M.I. (2018). Contributions to the knowledge of hard ticks (Acari: Ixodidae) in Colombia. Ticks Tick Borne Dis..

[70] Lerma L.S., Cogollo V.C., Velilla S.M., González I.R., Atehortua A.D.M., Peñuela D.F.C. (2019). First detection of *Candidatus* Rickettsia colombianensi in the State of Meta, Colombia. Rev. Habanera Cienc. Med..

[71] Cotes-Perdomo A., Santodomingo A., Castro L.R. (2018). Hemogregarine and Rickettsial infection in ticks of toads from northeastern Colombia. Int. J. Parasitol. Parasites Wildl..

[72] Santodomingo A., Sierra-Orozco K., Cotes-Perdomo A., Castro L.R. (2019). Molecular detection of *Rickettsia* spp., Anaplasma platys and Theileria equi in ticks collected from horses in Tayrona National Park, Colombia. Exp. Appl. Acarol..

[73] Cotes-Perdomo A.P., Oviedo Á., Castro L.R. (2020). Molecular detection of pathogens in ticks associated with domestic animals from the Colombian Caribbean region. Exp. Appl. Acarol..

[74] Estrada-Peña A. (2015). Orden Ixodida: Las garrapatas. Rev. IDEA-SEA.

[75] Osorno-Mesa E. (1942). Las garrapatas de la República de Colombia. Rev. Fac. Nac. Agron. Medellín.

[76] Martínez-Sánchez E.T., Cardona-Romero M., Ortiz-Giraldo M., Tobón-Escobar W.D., López D.M., Ossa-López P.A., Castaño-Villa G.J. (2020). Associations between wild birds and hard ticks (Acari: Ixodidae) in Colombia. Ticks Tick Borne Dis..

[77] Labruna B. (2009). Ecology of Rickettsia in South America. Ann. NY Acad. Sci..

[78] Sonenshine D.E., Roe R.M. (2013). Biology of Ticks.

[79] Kantsø B., Svendsen C.B., Jensen P.M., Vennestrøm J., Krogfelt K.A. (2010). Seasonal and habitat variation in the prevalence of Rickettsia helvetica in Ixodes ricinus ticks from Denmark. Ticks Tick Borne Dis..

[80] Estrada-Peña A., Bouattour A., Camicas J.L., Guglielmone A., Horak I., Jongejan F., Walker A.R. (2006). The known distribution and ecological preferences of the tick subgenus Boophilus (Acari: Ixodidae) in Africa and Latin America. Exp. Appl. Acarol..

[81] Schulze T.L., Jordan R.A. (1996). Seasonal and long-term variations in abundance of adult Ixodes scapularis (Acari: Ixodidae) in different coastal plain habitats of New Jersey. J. Med. Entomol..

[82] Adler G.H., Telford S.R., Wilson M.L., Spielman A. (1992). Vegetation structure influences the burden of immature Ixodes dammini on its main host, Peromyscus leucopus. Parasitology.

[83] Quintero J., Paternina L., Uribe A., Muskus C., Hidalgo M., Gil J., Cienfuegos A., Osorio L., Rojas C. (2017). Eco-epidemiological analysis of rickettsial seropositivity in rural areas of Colombia: A multilevel approach. PLoS Negl. Trop. Dis..

[84] Stańczak J., Racewicz M., Michalik J., Cieniuch S., Sikora B., Skoracki M. (2009). Prevalence of infection with Rickettsia helvetica in feeding ticks and their hosts in western Poland. CMI.

[85] Ioannou I., Chochlakis D., Kasinis N., Anayiotos P., Lyssandrou A., Papadopoulos B., Psaroulaki A. (2009). Carriage of *Rickettsia* spp., Coxiella burnetii and Anaplasma spp. by endemic and migratory wild birds and their ectoparasites in Cyprus. Clin. Microbiol. Infect.

[86] Spitalska E., Literak I., Kocianova E., Taragel’ova V. (2011). The importance of Ixodes arboricola in transmission of *Rickettsia* spp.. Anaplasma phagocytophilum, and Borrelia burgdorferi sensu lato in the Czech Republic, Central Europe. Vector Borne Zoonotic Diss..

[87] Hornok S., Kováts D., Csörgő T., Meli M.L., Gönczi E., Hadnagy Z., Hofmann-Lehmann R. (2014). Birds as potential reservoirs of tick-borne pathogens: First evidence of bacteraemia with Rickettsia helvetica. Parasites Vectors.

[88] Hornok S., Csörgő T., de la Fuente J., Gyuranecz M., Privigyei C., Meli M.L., Hofmann-Lehmann R. (2013). Synanthropic birds associated with high prevalence of tick-borne rickettsiae and with the first detection of Rickettsia aeschlimannii in Hungary. Vector-Borne Zoonotic Dis..

[89] Capligina V., Salmane I., Keišs O., Vilks K., Japina K., Baumanis V., Ranka R. (2014). Prevalence of tick-borne pathogens in ticks collected from migratory birds in Latvia. Ticks Tick Borne Dis..

[90] Lommano E., Dvořák C., Vallotton L., Jenni L., Gern L. (2014). Tick-borne pathogens in ticks collected from breeding and migratory birds in Switzerland. Ticks Tick Borne Dis..

[91] Eremeeva M.E., Dasch G.A. (2015). Challenges posed by tick-borne rickettsiae: Eco-epidemiology and public health implications. Public Health Front..

[92] Berthová L., Slobodník V., Slobodník R., Olekšák M., Sekeyová Z., Svitálková Z., Špitalská E. (2016). The natural infection of birds and ticks feeding on birds with *Rickettsia* spp. and Coxiella burnetii in Slovakia. Exp. Appl. Acarol..

[93] Parola P., Paddock C.D., Raoult D. (2005). Tick-borne rickettsioses around the world: Emerging diseases challenging old concepts. Clin. Microbiol. Rev..

[94] Telford III S.R., Parola P. (2007). Arthropods and rickettsiae. Rickettsial Diseases.

[95] Elfving K., Olsen B., Bergström S., Waldenström J., Lundkvist Å., Sjöstedt A., Nilsson K. (2010). Dissemination of spotted fever rickettsia agents in Europe by migrating birds. PLoS ONE.

[96] Walker D.H., Valbuena G.A., Olano J.P. (2003). Pathogenic mechanisms of diseases caused by Rickettsia. Ann. N. Y. Acad. Sci..

[97] Marrero M., Raoult D. (1989). Centrifugation-shell vial technique for rapid detection of Mediterranean spotted fever rickettsia in blood culture. Am. J. Trop. Med..

