# Supplementary material for: Molecular Detection of Rickettsia and Other Bacteria in Ticks and Birds in an Urban Fragment of Tropical Dry Forest in Magdalena, Colombia
Source: Life (Basel). 2023 Jan 4;13(1):145. doi: 10.3390/life13010145 (PMC9861851; doi:10.3390/life13010145)
Supplement: Supplementary file 1 [file life-13-00145-s001.zip › life-2102439-supplementary.pdf]

Table S1. Primers used for the molecular identification of ticks and Rickettsia.

Biological group	Gene	Amplicon (pb)	Primer	Primer sequence (5'-3')	Temperature (°C)	Reference
Ticks	COI	710	LCO1490	GGT CAA CAA ATC ATA AAG ATA TTG G	45	(Folmer et al., 1994)
			HCO2198	TAA ACT TCA GGG TGA CCA AAA AAT CA		
	16SrRNA	460	16S+1	CCG GTC TGA ACT CAG ATC AAG T	48	(Mangold et al., 1998)
			16s-1	GCT CAA TGA TTT TTT AAA TTG CTG T		
Rickettsia	GltA	401	CS-78	GCAAGTATCGGTGAGGATGTAAT	54	(Labruna et al., 2004a)
			CS-323	GCTTCCTTAAAATTCAATAAATCAG GAT		
	ompA	444	OMPA-F	CAC YAC CTC AAC CGC AGC	52	(Phan et al., 2011)
			OMPA-R	AAA GTT ATATTTCCT AAA CCY GTA TAA KTA TCR GC		
	Sca1	488	SCA1-F2	GGTGATGAAGAAGAGTCTC	49	(Anstead and Chilton, 2013)
			SCA1-R2	CTCTTTAAAATTATGTTCTAC		

Table S2. Blood samples from birds and ticks from vegetation processed by 16S metataxonomy amplicon sequencing.

Biological group	Species	month	Sample code
Birds	*Campylorhynchus griseus*	-	DLM1
	Crotophaga ani	-	DL151
	Furnarius leucopus	-	DLM2
	Cyclarhis gujanensis	-	DLM3
	Glaucidium brasilianum	-	DL241
	Hypnelus ruficollis	-	DLM4
	Melanerpes rubricapillus	-	DL88
	Leptotila verreauxi	-	DLM5
	Parkesia noveborasensis	-	DLM6
	Quiscalus lugubris	-	DLM7
	Saltator coerulescens	-	DLM8
Ticks	*Amblyomma dissimile*	Feb	ES2N
	Amblyomma dissimile	Feb	ES2L
	Amblyomma dissimile	Mar	ES3N
	Amblyomma dissimile	Mar	ES3L
	Amblyomma dissimile	Mar	ES3A
	Amblyomma dissimile	Abr	ES4N

Amblyomma dissimile	Abr	ES4L
Amblyomma dissimile	May	ES5N
Amblyomma dissimile	May	ES5L
Amblyomma dissimile	Jun	ES6N
Amblyomma dissimile	Jun	ES6L
Amblyomma dissimile	Jul	ES7N
Amblyomma dissimile	Jul	ES7L
Amblyomma dissimile	Ago	ES8N
Amblyomma dissimile	Ago	ES8L
Amblyomma dissimile	Oct	ES10L
Amblyomma dissimile	Oct	ES10N
Amblyomma dissimile	Nov	ES11L
Amblyomma dissimile	Nov	ES11N
Amblyomma dissimile	Dic	ES12N
Amblyomma dissimile	Dic	ES12L
Amblyomma dissimile	Ene	ES1N
Amblyomma dissimile	Ene	ES1L
Amblyomma dissimile	Ene	ES1A
Amblyomma dissimile	Feb	2ES2N
Amblyomma dissimile	Feb	2ES2L
Amblyomma dissimile	Feb	2ES2A

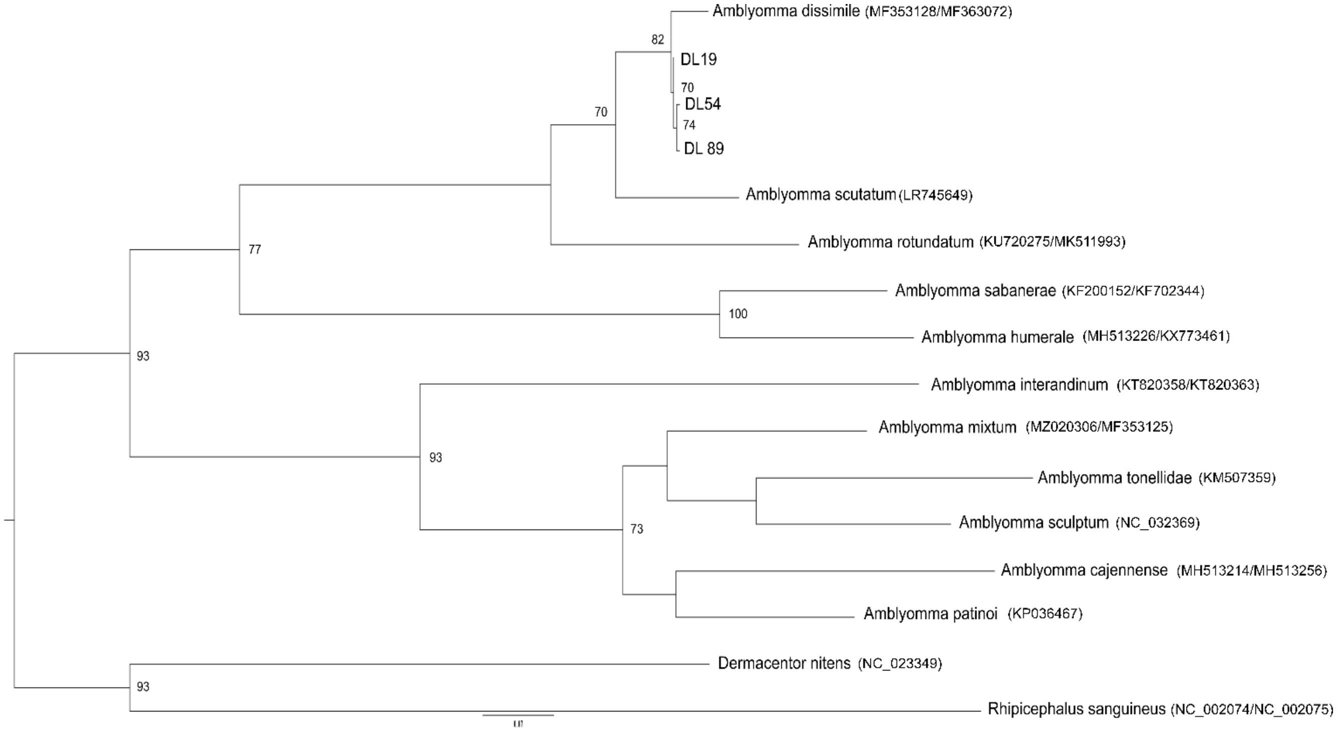

Figure S1. Phylogenetic reconstruction using maximum likelihood of the concatenated CO1 and 16SrDNA Amblyomma sequences obtained in this study and others downloaded from genbank with accession numbers included CO1/16S. Numbers on the nodes correspond to bootstrap values.

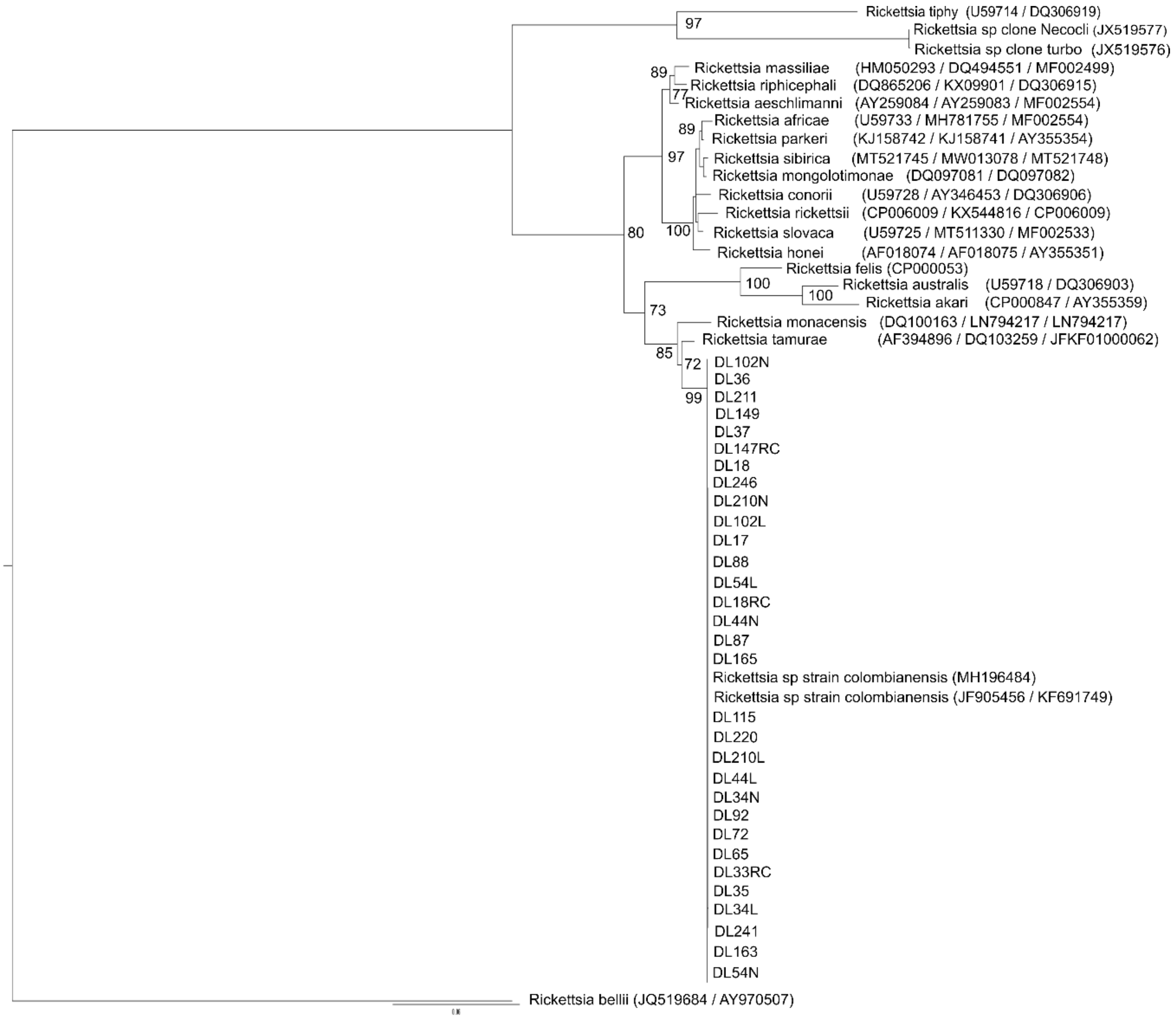

Figure S2. Phylogenetic reconstruction using maximum likelihood of concatenated GltA, OmpA and Sca1 genes for Rickettsia, including our sequences and sequences downloaded from genbank (with accession numbers included GltA/OmpA/Sca1). Numbers on nodes correspond to bootstrap values.

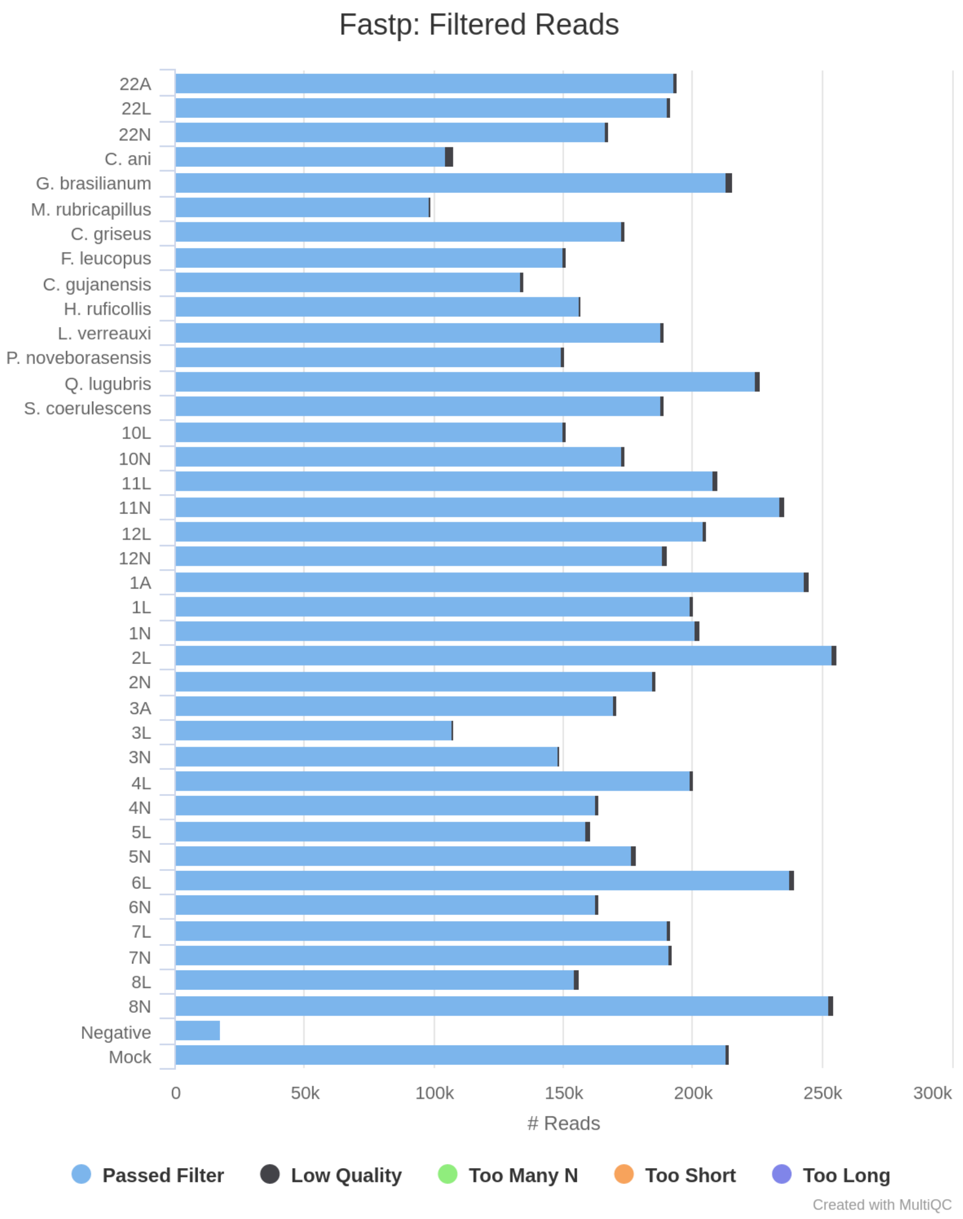

Figure S3. Number of reads for all samples. For the blood of birds' samples, the scientific name of the host where it was isolated is used. For the vegetation ticks´ samples, the number represents the month of collection (1-12 for all samples taken in 2021, 22 for the February 2022 samples), and the letter represents the ticks' life stage: (L)arva, (N)ymph, (A)dult.

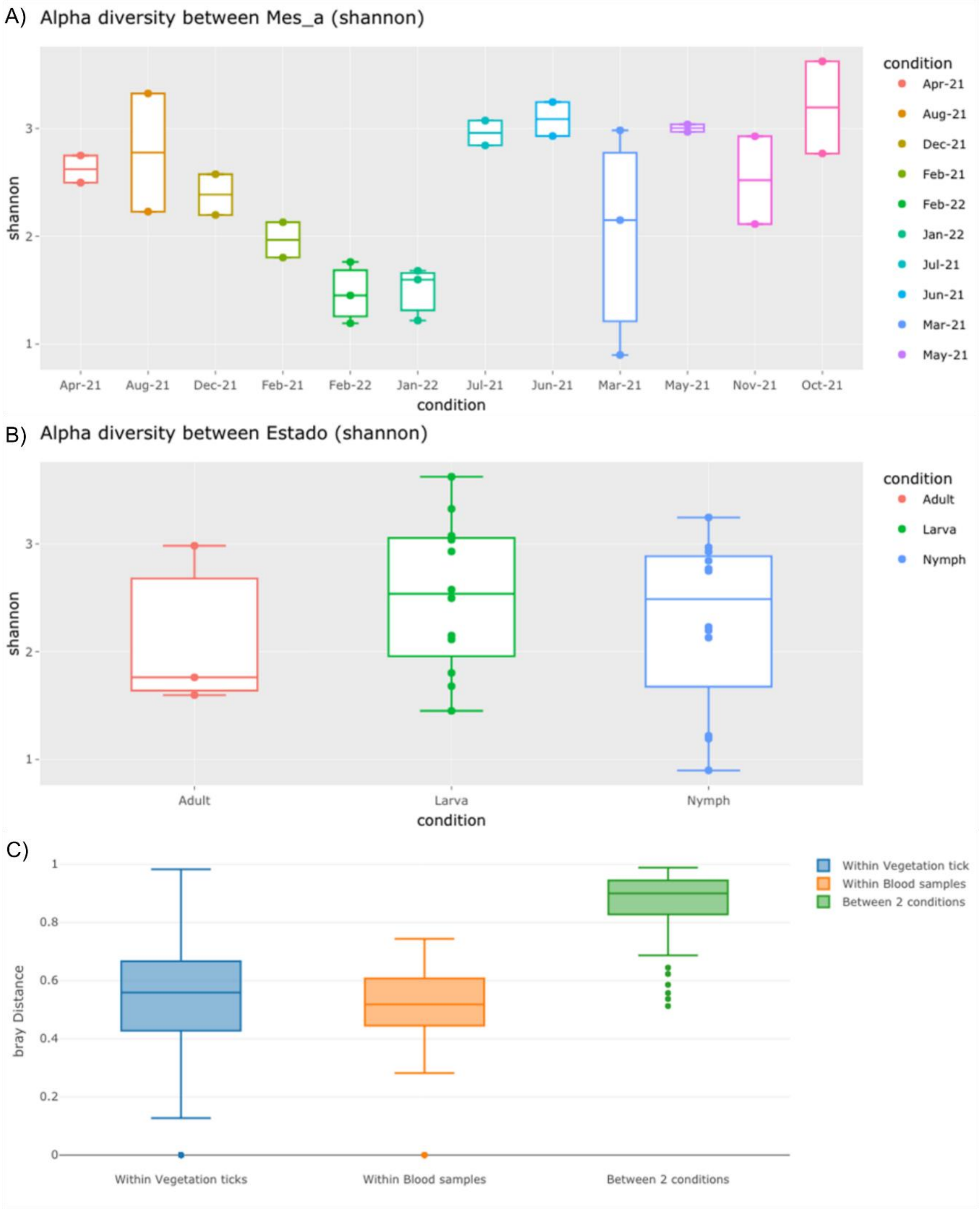

Supplementary Figure S4. Alpha diversity analysis based on Shannon index showed no significant differences (Kruskal-Wallis's rank sum test, $P < 0.05$) between A) Ticks life stage and B) vegetation ticks and blood samples. PERMANOVA test using Bray distance did showed significant beta diversity difference existing between vegetation ticks and blood samples ($P < 0.05$).
